# Use of In Vivo Optical Coherence Tomography (OCT) for Surgical Margin Assessment in Keratinocyte Carcinomas: A Systematic Review

**DOI:** 10.3390/cancers18101562

**Published:** 2026-05-12

**Authors:** Dana Bunevich, Monika Wojarska, Klaudia Kokot, Stanisław Antoniak, Amelia Barszczewska, Marcel Barbucha, Natalia Miszkin, Bogdan Jabłoński, Jerzy Jankau

**Affiliations:** 1 Students’ Scientific Circle of Plastic Surgery, Plastic Surgery Department, Medical University of Gdańsk, 80-210 Gdańsk, Poland; dana.bunevich@gumed.edu.pl (D.B.); s.antoniak@gumed.edu.pl (S.A.); ameliab@gumed.edu.pl (A.B.); marcelbarbucha@gumed.edu.pl (M.B.); natalia.miszkin@gumed.edu.pl (N.M.); bogdan.jablonski@gumed.edu.pl (B.J.); jerzy.jankau@gumed.edu.pl (J.J.); 2 Plastic Surgery Department, University Clinical Center in Gdańsk, 80-952 Gdańsk, Poland; 3 Scientific Circle of Neurotraumatology, Department of Emergency Medicine, Medical University of Gdańsk, 80-210 Gdańsk, Poland

**Keywords:** optical coherence tomography, OCT, keratinocyte carcinomas, skin cancer, surgical removal, Mohs surgery, presurgical, intraoperative, systematic review

## Abstract

**Background/Objectives:** Keratinocyte carcinomas (KCs), including squamous cell carcinomas (SCCs) and basal cell carcinomas (BCCs), are the most common malignancies worldwide. Accurate delineation of surgical margins is essential to ensure oncological control while preserving favourable aesthetic outcomes. Optical coherence tomography (OCT) is a non-invasive, real-time, high-resolution technique that may facilitate preoperative and intraoperative margin assessment. This systematic review aims to analyse existing evidence on the use of in vivo OCT in the assessment of surgical margins in KC. **Methods:** This systematic review was conducted in accordance with the PRISMA guidelines. The articles included were retrieved from the PubMed, Web of Science, Scopus and EBSCO databases. The search was limited to studies published between 2010 and November 2025. The inclusion criteria were the application of in vivo OCT as an adjunctive method in the surgical excision of KC. A total of 11 studies involving 303 patients met the inclusion criteria. OCT was used preoperatively, intraoperatively, combined pre- and intraoperatively and both pre-, intra- and postoperatively, with most studies focused on margin delineation by itself. **Results:** Agreement between OCT and histopathology ranges from 84% to 95.5%. Surgical benefits were observed in both conventional surgery and Mohs micrographic surgery, including reduction in MMS stages (1.89 to 1.23), fewer stages required, and narrower excision margins. **Conclusions:** Based on data analysis, it may be assumed that in vivo OCT may have potential in non-invasive margin assessment in the surgical treatment of KC. The available evidence is limited by the heterogeneity of protocols and devices and variability between lesion types, as well as the small sample sizes and limited comparability. Further research should focus on randomised multicentre trials involving large cohorts, cost–benefit analysis and evaluation of operator-dependent variability.

## Simple Summary

Keratinocyte carcinomas, including basal cell and squamous cell carcinomas, are the most common skin cancers worldwide, predominantly arising in sun-exposed anatomical sites. During surgical excision, precise margin assessment is crucial for preserving oncological safety and optimal aesthetic outcomes. Due to current evidence, both classic surgery and Mohs micrographic surgery are recommended for surgical treatment of KC. Optical coherence tomography is a non-invasive imaging technique that provides real-time, high-resolution images of skin structure and may facilitate margin assessment of KC. This systematic review analysed studies that evaluated whether in vivo optical coherence tomography can be an adjunctive tool for margin delineation. Analysis of 11 studies involving 303 patients suggests potentially beneficial results, in particular, fewer MMS stages, less excision of healthy tissue and shorter procedure time. However, larger clinical trials are still required to investigate the role of optical coherence tomography in clinical practice.

## 1. Introduction

Keratinocyte carcinomas represent the most common malignancies in fair-skinned populations. Men are affected more frequently than women. KC is also age-related; the older population is more susceptible [1]. The term “keratinocyte carcinomas” comprises basal-cell carcinomas (BCCs) and squamous-cell carcinomas (SCCs). BCC is the most prevalent type of malignant skin neoplasm, accounting for approximately 75–80% of cases, whereas SCC accounts for approximately 15–20% [2], with recent studies suggesting a relative increase in the proportion of SCC [3,4]. This is the trend observed in studies conducted by HW Rogers et al. (2015) and Harvey I et al. (1989) and is related to chronic, cumulative UV exposure over decades [3,5]. Despite their low mortality, KCs are associated with substantial morbidity [1,6]. Cutaneous SCC is primarily associated with advanced age (mean age of 70), with over 80% of cases occurring in patients aged 60 and older. Men are affected twice as often as women worldwide. While the mortality rate for BCC is 0.87 to 0.97, for SCC it is 1.17 to 1.30 [1].

KCs are predominantly caused by UV exposure [6,7]. In this regard, there is a strong correlation between the localisation of KC and sun-exposed areas such as the so-called H-zone, associated with the incidence of high recurrence risk of KC, in particular the nose, eye area, cheek, temple, forehead, lips and nasolabial folds [8,9,10]. Surgical excision remains the standard treatment for high-risk KC, with current guidelines recommending resection margins of 5–15 mm for high-risk BCC and 6–10 mm for high-risk SCC [11,12,13]. Excision with guideline-recommended margins may lead to excessive tissue loss and require reconstructive surgery to preserve both functional integrity and aesthetic outcomes.

Mohs micrographic surgery (MMS) represents the gold standard for the management of high-risk BCC and SCC [11]. This technique involves the stepwise, layer-by-layer excision of tumour margins with intraoperative histopathological assessment. Although this method minimises excision margins and demonstrates a low recurrence rate compared to classic surgery, MMS in Europe is underused due to its cost, time-consuming procedure and limited number of trained centres [14,15,16]. Research conducted by Essers et al. (2006) showed the total average costs/patient were higher in the MMS than in the SSE group (primary treatment of BCC: €1248 vs. €1044); recurrent BCC: €1289 vs. €1234 [17]. This may explain why a systematic bias has developed in favour of patients who do not choose this treatment method. Despite the nominal price increases observed over the last two decades, when adjusted for inflation, the cost gap between MMS and SSE remains remarkably consistent with the 2006 findings.

According to an international survey by Christine P. Lin et al. (2025), the main barriers to the expansion of Mohs micrographic surgery include limited public awareness (41.1%), inadequate infrastructure (40.5%), and insufficient reimbursement (31.0%) [18]. Additionally, inadequate reimbursement systems across many countries fail to cover the high costs of specialised laboratory equipment and personnel required for the procedure [18].

When designing studies, it is important to consider differences between MMS and conventional surgery. For MMS, retrospective or non-randomised studies conducted at referral centres and focusing primarily on the treatment of high-risk tumours, may be more feasible, whereas studies of conventional surgery are more frequently prospective and involve more diverse patient populations, including both low- and high-risk patients.

**Figure 1:**
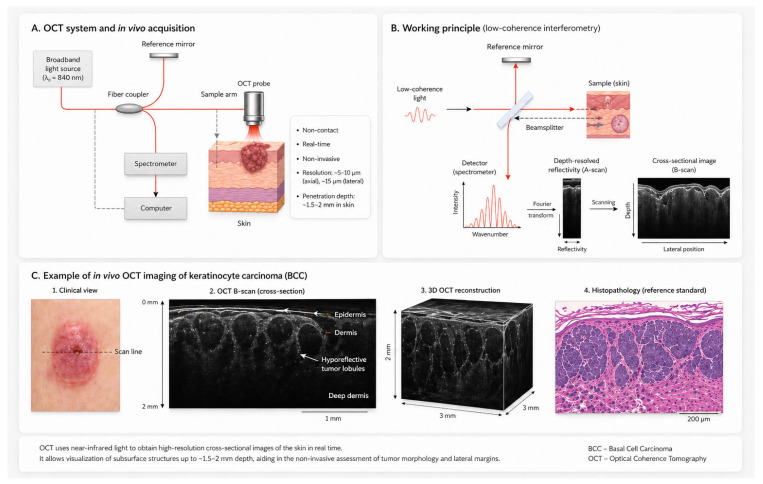
OCT: principle and in vivo application for skin KC margin assessment.

Optical coherence tomography (OCT) is a non-invasive technique with significant potential for preoperative assessment of KC margins [19,20]. It provides resolution of approximately 5–10 µm, lateral <7.5–15 µm, and penetration depth up to 1.5–2 mm [21]. OCT stands out due to its high speed, high resolution (3–15 μm), high signal sensitivity, and penetration depth of 1–3 mm, with a scanning field of 6 × 6 mm, making it proper for detecting KC. It serves as a supplementary diagnostic tool, allowing for preliminary diagnoses before biopsy. As OCT technology has advanced, it has disclosed more structural details of the skin, increasing diagnostic accuracy. These details aid in identifying BCC subtypes and distinguishing AKs from SCCs and normal skin [22]. Figure 1 illustrates the OCT scanning process.

A more advanced modality, line-field confocal OCT (LC-OCT), has also been developed. LC-OCT enables high-resolution, three-dimensional imaging—often described as a ‘virtual biopsy’—with a penetration depth of approximately 500 µm and lateral and axial resolutions of approximately 1.3 µm and 1.2 µm, respectively [19,23]. In some studies, OCT has demonstrated sensitivity and specificity approaching those of conventional histopathology [24]. A study conducted by Donelli C. et al. (2023) proposes key image markers for each type of lesion, individual LC-OCT features that each have a histopathological correlation and are typically associated with i.a. SCC or BCC in traditional histopathology [25]. The presence of hypo-reflective roundish lobules, clefting, and hyperreflective stroma and highly hypo-reflective areas corresponding to dilated vessels in LC-OCT supported the hypothesis of BCC. When compared with dermoscopy, using histopathology as the gold standard, LC-OCT showed superior sensitivity, specificity, and diagnostic accuracy. The sensitivity, specificity, and diagnostic accuracy for LC-OCT were 0.99 (CI 0.97–1.00), 0.90 (CI 0.84–0.94), and 0.96 (CI 0.93–0.97), respectively, compared with 0.97 (CI 0.94–0.99), 0.43 (CI 0.36–0.51), and 0.77 (CI 0.72–0.81) for dermoscopy [25].

Additionally, by offering detailed structural information, LC-OCT may help assess cosmetic outcomes and skin regeneration following radiotherapy, which is of increasing interest given the potential for long-term aesthetic and functional sequelae [26].

These features suggest that OCT may serve as an adjunct technology in diagnosis, therapeutic decision-making, preoperative margin assessment, reduction in the number of MMS stages, and the reduction in excision margins while maintaining complete tumour removal.

Based on currently available data, there are only a few studies demonstrating the effect of OCT in vivo on margin assessment of keratinocyte carcinomas. Previous studies have focused primarily on diagnostic accuracy in the detection of basal cell carcinoma.

For example, Cheng et al. (2015) conducted a systematic review that included 31 original studies examining the diagnostic use of OCT in the context of BCC, covering parameters such as subtyping and the monitoring of the effects of non-surgical treatment [27]. Similar to the previous study, the research questions addressed in the review by Ferrante di Ruffano et al. (2018) were whether OCT could improve the diagnostic accuracy of BCC and SCC compared to direct visual examination and dermatoscopy [28].

This systematic review differs from the existing literature in that the scope is narrowed and focused on the use of OCT in vivo for margin assessment of keratinocyte carcinomas during or prior to their excision.

This systematic review addressed the following research questions:

1. Can in vivo OCT serve as an adjunctive method for preoperative and intraoperative margin delineation of keratinocyte carcinomas?
2. What level of concordance with histopathology does in vivo OCT provide?
3. Does preoperative or intraoperative OCT help reduce the number of stages in Mohs micrographic surgery (MMS) and the total duration of the procedure?
4. How does the use of in vivo OCT affect the size of resection margins?
5. What limitations of the technique currently exist that hinder its introduction into clinical practice?

These issues are addressed in Section 3 and Section 4 through a summary of objective measures (e.g., sensitivity, coefficients of agreement, reduction in disease stage) and a critical assessment of study heterogeneity.

The aim of this systematic review is to synthesise and critically evaluate current literature on the use of in vivo OCT for surgical margin assessment in keratinocyte carcinomas. Although OCT has shown promising preliminary results in this context, the currently available evidence remains limited. The existing studies are constrained by the lack of large prospective multicentre trials, standardised imaging protocols, and long-term follow-up of large patient cohorts. Therefore, this review focuses on assessing the potential clinical applicability of the method based on current evidence and identifying gaps that require further investigation.

## 2. Materials and Methods

### 2.1. Study Design

This systematic review was conducted in accordance with the Preferred Reporting Items for Systematic Reviews and Meta-analysis (PRISMA) guidelines. Prisma 2020 Checklist has been included as Supplementary Material. The protocol was prospectively registered in the PROSPERO database (registration ID: CRD420261351793). The articles included in this review were retrieved from the PubMed, Web of Science, Scopus and EBSCO databases.

The time frame for the literature search was set from 2010 to November 2025 to reflect both the increased scientific output on OCT and ensure the inclusion of the most recent publications. The following search query was used: (“OCT” OR “optical coherence tomography” OR “line-field confocal OCT” OR “LC-OCT”) AND (“NMSC” OR “non-melanoma skin cancer” OR “BCC” OR “basal cell carcinoma” OR “SCC” OR “squamous cell carcinoma” OR “keratinocyte carcinomas” OR “skin cancer”) AND (“presurgical” OR “surgical removal” OR “Mohs” OR “intraoperative” OR “margin assessment” OR “margin delineation”) A formal risk-of-bias assessment was performed using the Newcastle-Ottawa Scale (NOS). Two reviewers independently evaluated the methodological quality of the included studies across three domains: selection, comparability, and outcome. Any discrepancies were resolved through discussion.

### 2.2. Eligibility Criteria

The primary inclusion criterion was the use of in vivo OCT as an adjunctive method in the surgical excision or margin assessment of KC. Exclusion criteria involved: (a) use of ex vivo OCT; (b) exclusive use of OCT as a diagnostic method; (c) use of OCT without marking tumour margins; (d) use of OCT as an adjunct to surgical treatment of melanoma; (e) removal of the lesion using other non-surgical methods (e.g., laser therapy); (f) use of combined techniques (e.g., OCT + RCM); (g) abstracts, case reports, conference papers, letters, and editorial articles; and (h) articles written in languages other than English.

### 2.3. Study Selection

Following an initial search, a total of 657 articles were identified. By excluding all duplicate content, 311 articles were left. Mendeley Software (Reference Manager Version 2.130.2) was used to exclude duplicate results. In the next step, to avoid the risk of incomplete deduplication, the articles were manually verified to definitively eliminate duplicates. Two independent reviewers screened the studies based on their titles to determine which ones were eligible for abstract analysis. Following an initial assessment of the articles based on their titles alone, 55 articles remained. Then the abstracts of these articles were analysed by the same two reviewers, and, based on this, 36 articles were included for full-text analysis. As a result of the full-text analysis and based on compliance with the inclusion criteria, 11 articles involving a total of 303 patients were included in the final review.

Any discrepancies at any stage of the selection process were first resolved through discussion between the two reviewers until a consensus was reached. In the event of a disagreement, the final decision was made by a third reviewer (the arbitrator). Cohen’s kappa coefficient demonstrated substantial agreement between reviewers (κ = 0.82).

**Figure 2:**
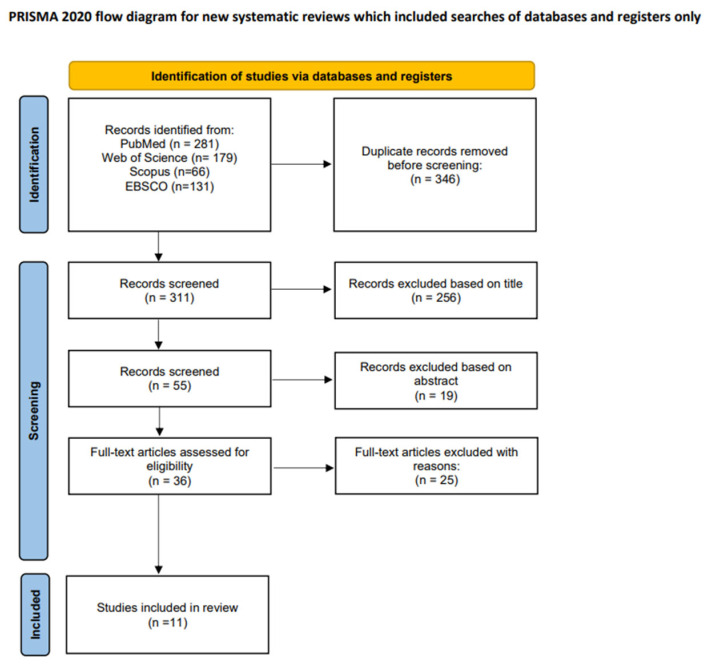
PRISMA flowchart.

Any discrepancies were resolved through consensus. The study selection process is presented in a flow diagram, Figure 2.

## 3. Results

A complete list of the studies included in the review is presented and analysed in Table 1 and Table 2. OCT technical modalities are presented in Table 3.

The methodological quality of the included studies, assessed using the Newcastle-Ottawa Scale (NOS), ranged from moderate to low risk of bias. Four studies were classified as low risk of bias (7–9 stars), while the remaining studies were of moderate quality (4–6 stars). No studies were rated as high risk of bias. The most common limitations were related to comparability, particularly the lack of adjustment for confounding variables, as well as small sample sizes and retrospective study designs. Higher-quality studies were typically characterised by larger cohorts and more rigorous outcome assessment. Detailed results of the quality assessment are presented in Table 4.

### 3.1. Type of Cancer

The vast majority of articles focused on BCC research. Seven studies focused on BCC, three on a combined cohort of BCC and SCC, and one on SCC exclusively.

### 3.2. Type of Surgery

Conventional surgery and Mohs surgery were represented in comparable proportions: five studies involving 151 patients described the use of OCT in combination with Mohs surgery, whilst five other studies involving 129 patients described the technique using conventional surgery. One study, involving 23 patients, used histopathological examination. OCT was used preoperatively in eight studies, intraoperatively in one study, both pre- and intraoperatively in one study, and pre-, intra-, and postoperatively in one study.

### 3.3. The Use of OCT

Nine studies focused on margin assessment. Of these, Mohs surgery was used in four articles involving a group of 91 patients, conventional surgery was used in four articles involving a group of 57 patients, and histopathological examination was used in one article involving 23 patients.

Two studies covered both the diagnostic use of OCT and the delineation of margins. Of these, one—describing 71 patients—used conventional surgery, whilst the other one—involving a group of 22 patients—used Mohs surgery.

### 3.4. The Type of OCT

Nine studies used conventional OCT, one used LC-OCT, and one used SD-OCT.

Table 5 presents a data summary showing the distribution of the 11 studies included in the review by cancer type and OCT modality.

### 3.5. Study Results

Among the objectively assessable data reported in the studies, a high level of agreement between OCT results and conventional histopathology was observed: 86.6% (52/60 data points) [30], 95.5% (κ = 0.89) [20], and 84% correct lateral margins [32].

Sensitivity and specificity varied depending on the type of neoplasm and the device used. In conventional OCT for BCC, diagnostic performance for BCC appeared generally high, whereas for SCC the reported results were more variable and, in some studies, fell below thresholds considered adequate for reliable clinical correlation (e.g., 98% sensitivity and 96% specificity for BCC, 96% sensitivity and 97% specificity for SCC [31]; 92.5% sensitivity and 68.8% specificity for SCC [33]). These results suggest that there is heterogeneity in diagnostic accuracy, particularly in SCC, and that the conclusions are based on very limited and recent preliminary data.

Regarding the timing of OCT use during surgical intervention, most studies focused on the preoperative application of OCT. OCT showed high agreement with histopathology, reaching up to 95.5% [20]. In some studies, preoperative use of OCT was associated with narrower excision margins and a reduced number of Mohs stages during MMS [36]. Limited evidence was available for intraoperative use of OCT. One study suggested that OCT may enable precise tumour mapping [33]. In one study, a combination of pre- and intraoperative OCT use was applied and suggested that OCT has the potential to accurately define margin borders before MMS [37]. One study used OCT pre-, intra-, and postoperatively, and reported that OCT appears to be a promising tool in the diagnosis of skin pathologies [31]. Regarding the timing of OCT use, the reported sensitivity and specificity showed promising results; however, stronger conclusions require larger cohort studies with bigger study populations.

In the only study to use LC-OCT technology, the ability to subtype BCC was fairly moderate, with a high level of stratification of high-risk lesions (66.7%, κ = 0.49 for subtyping and 95.2%, κ = 0.88, AUC = 0.93 for risk stratification) [19]. These results are promising; however, it is worth noting that they are based on a single study. To be able to draw stronger conclusions regarding the usefulness of LC-OCT in this context, more evidence and larger cohort studies are required.

The study highlighted the direct surgical benefits of using OCT in both conventional surgery and Mohs micrographic surgery (MMS). In a case–control study, preoperative LC-OCT mapping significantly reduced the mean number of MMS stages from 1.89 ± 1.05 to 1.23 ± 0.43 (*p* = 0.007), with a significantly lower risk of requiring more than one stage (odds ratio 0.3, 95% CI 0.1–0.8, *p* = 0.032) [19]. In another study, OCT-guided assessment led to a reduction in the number of MMS stages in 50% of cases (11/22 patients) [20]. In a small pilot series, single-stage excision was achieved in 8 out of 10 cases of BCC [36]. Furthermore, margins determined using OCT were on average 0.4 ± 1.1 mm narrower than those assessed clinically by the surgeon [35]. However, most of these results come from relatively small, non-randomised studies with significantly limited control groups, and larger prospective controlled trials are needed to confirm these potential benefits.

The VivoSight OCT system, which is the conventional and most widely used system, provides high-resolution imaging: axial <5–10 µm, lateral <7.5–15 µm, and depth up to 1.5–2 mm. OCT demonstrates high precision for lesions with infiltration depth ≤ 1 mm, whereas its accuracy decreases in lesions with greater depth ≥ 1 mm. The correlation between effectiveness and infiltration depth may be a limitation in this treatment method and be reflected in a reduced correlation between OCT measurements and histopathological findings in tumour depth assessment of infiltrative lesions [29,34].

## 4. Discussion

This systematic review provides updated synthesis of the available evidence on the use of OCT in the surgical management of keratinocyte carcinomas. As a non-invasive imaging technique for KC, OCT aligns with current trends toward minimising invasiveness in oncologic diagnostics and treatment. The reviewed literature, although limited, suggests clinically relevant findings regarding its potential role in both diagnosis and surgical planning. Key areas of interest included the diagnostic performance of OCT, its accordance with histopathology, sensitivity and specificity, as well as its impact on surgical outcomes, including reduction in excision margins and the number of Mohs micrographic surgery (MMS) stages. A total of 11 articles included in the review indicate that in vivo OCT may be useful not only for diagnosis but also for pre- and intraoperative assessment of margins in keratinocyte carcinomas. These findings can be broadly categorised into two domains: diagnostic utility and direct surgical impact.

In terms of its diagnostic performance, OCT represents a middle ground between dermatoscopy and histopathological examination. Most studies indicate good superficial demarcation of the margins [29] and high rates of agreement with histopathology [19,20,30,32,33,34], as well as sensitivity and specificity rates reported [19,31,33]; however, it is limited by the depth of penetration of the device and may not be applicable for infiltrative types [29,34].

OCT and RCM are frequently compared in the context of their use in the diagnosis and margin delineation of KC. While RCM offers high surface resolution and good visualisation of cellular and subcellular structures (approximately 1 µm), its image penetration depth is shallower (200–300 µm) than that of conventional OCT (1.5–2 mm). An additional advantage of OCT is a wider scanning field (up to several mm compared to 0.5 × 0.5 mm in RCM) as well as a high image acquisition rate. In this context, the use of OCT appears more justified for margin delineation, as it captures a larger field of analysis of the lesion and has the potential to accelerate the intraoperative assessment process. A certain compromise technique is LC-OCT, which occupies an intermediate position between OCT and RCM, combining resolution close to that of RCM (1–2 μm) with a penetration depth of up to 500 μm [38,39,40,41].

Table 6 provides a brief comparative analysis of the methods described above.

Agreement with histopathology was particularly strong for lateral margin assessment, whilst lower concordance was reported for other parameters such as tumour depth. While OCT demonstrated good accuracy in measuring tumour depth in lesions ≤ 1 mm, it tended to underestimate invasion depth in thicker lesions (>1 mm) and may be a limitation [29].

The usefulness of OCT also appears to differ between BCC and SCC. In BCC, characteristic morphological patterns allow relatively reliable visualisation of tumour structures. In contrast, in SCC the presence of hyperkeratosis and deeper infiltration often limits image clarity, reducing diagnostic confidence. This distinction highlights both the advantages and limitations of OCT depending on tumour type.

In most studies, the direct impact of using OCT in a surgical context was promising. The aim of some studies was to compare the clinical margins of the tumour with those determined by OCT. False-positive findings were described in only one study [20], which occasionally resulted in enlargement of the resection margin. This may be attributable to OCT imaging artefacts. While larger studies are required to better define the frequency and clinical relevance of such artefacts, the available evidence indicates that the potential benefits of OCT—including narrower margins and fewer MMS stages—likely outweigh this limitation. Indeed, studies using OCT in MMS have demonstrated a reduction in the number of resection stages, a shorter operative time, and improved prediction of subsequent stages [19,20,35].

Due to the heterogeneity among the studies, it is not possible to draw definitive and generalised conclusions regarding the effects of OCT in vivo for surgical margin assessment of KC. The observed variability in the results may be attributed to the experimental nature of the technique; the lack of standardised research protocols; differences related to device types (e.g., conventional time-domain, spectral-domain or line-field confocal OCT) and technical device settings and scanning type (freehand scanning, point-by-point scanning or multislice scanning); variations in histological types of lesions (e.g., nodular and infiltrative types of BCC); varying disease stages; and anatomical location.

Given the generally small number of studies, most of the available research has focused specifically on BCC; therefore, it currently limits the generatability of the findings to KC as a group.

### 4.1. Strengths and Limitations of the Study

This systematic review has several limitations that should be considered when interpreting the findings.

First, the search strategy excluded studies evaluating combined imaging modalities (e.g., OCT combined with reflectance confocal microscopy). This approach was intended to reduce confounding effects related to multimodal imaging; however, it may have led to the exclusion of relevant evidence and thus limited the comprehensiveness of the review.

Second, the review is subject to potential language bias, as only studies published in English were included.

Third, the overall methodological quality of the included studies was predominantly moderate, with common limitations including small sample sizes and limited comparability between study groups. These factors may reduce the strength and generalisability of the conclusions.

Furthermore, most available studies focused primarily on basal cell carcinoma, with fewer studies addressing squamous cell carcinoma. Although this reflects the epidemiological distribution of keratinocyte carcinomas, it may limit the generalisability of the findings to KC as a whole.

Comparisons between Mohs micrographic surgery and conventional surgical excision should be interpreted with caution due to inherent differences in patient selection and clinical context. MMS is typically performed in high-risk cases, whereas conventional surgery often includes a more heterogeneous patient population, which may introduce bias in outcome comparisons.

In addition, the clinical applicability of OCT for margin mapping may be limited by operator-dependent factors. Accurate interpretation of OCT images and precise correlation with the physical location of the lesion require experience and technical skill, including visual-motor coordination. This may represent a barrier to widespread implementation in routine clinical practice.

Finally, technical limitations of OCT, including limited penetration depth, may reduce its accuracy in assessing deeper or infiltrative tumours.

### 4.2. Future Directions

In the context of future research, a cost-effectiveness analysis—covering aspects such as the cost of the procedure, staff training requirements, and the duration of the operation—may be both justified and practically relevant. It should also consider potential savings resulting from a possible reduction in the number of reoperations due to more accurate margin assessment and more precise surgical planning, which could shorten overall procedure time.

## 5. Conclusions

The data analysis suggests that in vivo OCT may have potential for non-invasive diagnosis and shows promise in the surgical treatment of keratinocyte carcinomas. Initial studies have shown that the technique may reduce possible benefits, the number of MMS stages and the excision of healthy tissue. However, the heterogeneity in protocols and devices, the lack of large randomised multicentre trials, limited long-term follow-up data, and the lack of cost-effectiveness analysis represent major barriers to the incorporation of this technique into clinical practice.

## Figures and Tables

**Table 1:** Articles included in the systematic review.

Authors	Surgery	No. of Patients	OCT Use	Cancer	OCT Timing	Quantitative Outcomes	Device
Coleman AJ. et al., 2013 [29]	Biopsy without complete excision	23	DA	BCC, SCC	Pre-op	Depth difference (OCT vs. histology): −0.54 ± 1.14 mm for lesions > 1 mm (significant underestimation) −0.17 ± 0.3 mm for lesions < 1 mm	VivoSight
Holm KBE et al., 2023 [30]	Classic removal	10	MD	BCC	Pre-op	Agreement with histopathology: 86.6% (52/60 DMta points)	VivoSight Dx
Jerjes W. et al., 2021 [31]	Classic removal	72	MD, DM	BCC, SCC	Pre-op, intra-op, post-op	1. In vivo OCT—diagnosis of SCC: Sensitivity: 96% Specificity: 97% 2. In vivo OCT—diagnosis of BCC: Sensitivity: 98% Specificity: 96%	EX1301 OCT Microscope V1.0
Alawi SA. et al., 2013 [32]	Classic removal	18	MD	BCC	Pre-op	1. Correct lateral margins identified by OCT: 84% of cases (16/18) 2. Surgical margins: Never fell below the margin determined by OCT	VivoSight
Sunny SP. et al., 2019 [33]	Classic removal	14	MD	SCC	Intra-op	Sensitivity: 92.5% Specificity: 68.8% 1. Agreement with histopathology: 0.59 2. High-grade dysplasia detection: 100%	SD-OCT (Spectral Domain OCT)
Lucia Pelosini et al., 2013 [34]	Classic removal	15	MD	BCC	Pre-op	1. Correlation with histopathology: x-axis: r = 0.80 y-axis: r = 0.66 Depth: r = 0.43 2. Visibility of margins: Horizontal (x-axis): 3/15 Horizontal (y-axis): 6/15 3. Depth measurable in: 9/15	VivoSight
Paradisi A et al., 2024 [19]	Mohs	60	MD, DM	BCC	Pre-op	1. BCC subtype agreement: 66.7% (κ = 0.49) 2. Low/high-risk stratification: 95.2% (κ = 0.88, AUC = 0.93) 3. Sensitivity: 87.5% (95% CI: 67.6–97.3) 4. Specificity: 98.8% (95% CI: 93.7–100) 5. Mean Mohs stages (study vs. control): 1.23 vs. 1.89 6. Need for >1 stage: 22.7% vs. 53.7% 7. Need for >3 stages: 0% vs. 22% 8. Chance of shortening the operation: 5 times higher (OR = 0.2)	LC-OCT (Line-field confocal OCT)
Akella SS. et al., 2024 [20]	Mohs	22	MD	BCC	Pre-op	1. Agreement with histopathology: 95.5% (21/22, κ = 0.89, *p* < 0.01) 2. Reduction in Mohs stages: 50% (11/22 patients) 3. False-positive results: 10% (2/22) 4. Correct identification of tumour absence: 86% (6/7)	VivoSight Dx
Wang KX. et al., 2013 [35]	Mohs	52	MD	BCC	Pre-op	1. OCT margins: On average 0.4 ± 1.1 mm smaller than surgeon’s clinical assessment 2. Mohs defect: 1.4 ± 1.1 mm larger than OCT margin 3. Surgeon’s assessment: 1.0 ± 1.2 mm too wide 4. False positives: 0% 5. Prediction of need for second Mohs stage: 100% correct	VivoSight
De Carvalho N. et al., 2018 [36]	Mohs	10	MD	BCC	Pre-op	1. Single-stage excision achieved: 8/10 BCC cases 2. Margin enlargement after OCT: 4/10 cases (reducing need for additional MMS stages)	VivoSight Dx
Rosales Santillan M et al., 2025 [37]	Mohs	7	MD	BCC	Pre-op, intra-op	1. Agreement with surgeon-defined lateral margins: 6/8 cases 2. OCT detected tumour beyond surgeon-defined margins: 2/8 cases	VivoSight

Legend: OCT—Optical Coherence Tomography, MD—Margin Delineation, DA—Depth Assessment, DM—Diagnostic Method, BCC—Basal Cell Carcinoma, SCC—Squamous Cell Carcinoma, Mohs—Mohs Micrographic Surgery.

**Table 2:** Qualitative (subjective/clinical) findings.

Authors	Key Qualitative Findings
Coleman AJ. et al. 2013 [29]	(a) Accurate for superficial BCC dimensions (b) Detects peripheral palisading (nodular BCC)
Holm KBE et al. 2023 [30]	Useful for pre-/intraoperative BCC MD
Jerjes W. et al. 2021 [31]	(a) Promising diagnostic tool for skin cancers (b) High accuracy for BCC and SCC
Alawi SA. et al. 2013 [32]	(a) OCT-guided margins can be reduced safely (b) Maintains complete excision rate (c) Perpendicular point-by-point scanning is not recommended
Sunny SP. et al. 2019 [33]	(a) Detects tumours in clinically normal tissue (b) Enables accurate tumour field mapping
Lucia Pelosini et al. 2013 [34]	(a) Strong correlation for lateral margins (b) Weak correlation for invasion depth
Paradisi A et al. 2024 [19]	-
Akella SS. et al. 2024 [20]	(a) Reduces number of Mohs stages (b) May reduce tissue excision (c) Potential to avoid surgery in OCT-negative lesions
Wang KX. et al. 2013 [35]	-
De Carvalho N. et al. 2018 [36]	-
Rosales Santillan M et al. 2025 [37]	(a) Accurately assesses lateral BCC margins (b) Detects tumour beyond clinical margins

**Table 3:** Overview of OCT systems used across the included studies.

Device/System	Depth Limit	Lateral Resolution	Axial Resolution	No. of Studies	No. of Patients
VivoSight (Michelson Diagnostics)	1.2–2.0 mm	<7.5–15 μm	<5–10 μm	7	162
VivoSight Dx (Michelson Diagnostics)	1.0–1.5 mm	<7.5 μm	<5 μm	3	92
EX1301 OCT Microscope V1.0	1.5 mm	<10 μm	<10 μm	1	72
Spectral Domain OCT (SD-OCT)	up to 2.0 mm	<15.0 μm	<7.0 μm	1	14
Line-field confocal OCT (LC-OCT)	≈500 μm	≈1.3 μm	≈1.1–1.2 μm	1	60

**Table 4:** Risk of bias assessment of included studies using the Newcastle-Ottawa Scale (NOS).

Study (Author, Year)	Selection (Max 4★)	Comparability (Max 2★)	Outcome (Max 3★)	Total (Max 9★)	Risk of Bias
Coleman, 2013 [29]	★★★	★	★★	6	Moderate
Holm, 2023 [30]	★★★	★	★★	6	Moderate
Jerjes, 2021 [31]	★★★★	★★	★★★	9	Low
Alawi, 2013 [32]	★★★	★	★★	6	Moderate
Sunny, 2019 [33]	★★★	★	★★	6	Moderate
Pelosini, 2013 [34]	★★★	★	★★	6	Moderate
Paradisi, 2024 [19]	★★★★	★★	★★★	9	Low
Akella, 2024 [20]	★★★★	★★	★★★	9	Low
Wang, 2013 [35]	★★★★	★★	★★★	9	Low
De Carvalho, 2018 [36]	★★★	★	★★	6	Moderate
Rosales Santillan, 2025 [37]	★★	★	★★	5	Moderate

**Legend:** 4–6 stars = moderate risk; 0–3 stars = high risk.

**Table 5:** Distribution of Included Studies and Patients According to Cancer Type and OCT Modality.

Cancer Type	Conventional OCT	SD-OCT	LC-OCT	Total Studies	Total Patients
BCC only	6	0	1	7	166
SCC only	0	1	0	1	14
BCC + SCC (mixed)	3	0	0	3	113
Total	9	1	1	11	303

**Table 6:** Comparative performance of in vivo optical coherence tomography (OCT/LC-OCT), reflectance confocal microscopy (RCM), dermoscopy, and histopathology in the diagnosis and surgical margin assessment of keratinocyte carcinomas [28,29,42,43,44,45].

Parameter	RCM	OCT	Dermoscopy	Histopathology
Invasiveness	Non-invasive	Non-invasive	Non-invasive	Invasive
Resolution	~1–2 µm (near histological)	Lateral <7.5–15 µm, axial <5–10 µm	Surface-related	Subcellular
Depth	~200–300 µm (superficial dermis)	1.5–2 mm	No depth	Full tissue thickness
Sensitivity (BCC)	~92–97%	~95–99%	~91%	~100%
Specificity (BCC)	~85–95%	~75–90%	~43–95%	~100%
Diagnostic accuracy	~90–95%	~96%	~77–95%	~100%
Processing time	Moderate (min, requires expertise)	Fast (min)	Fast (min)	Slow (h–days)
Costs	High	Moderately high	Low	High
Assessment of surgical margins	Good for lateral margins; limited for depth	Good correlation for lateral margins (84–95.5% agreement); limited depth assessment	Limited (superficial only)	Gold standard
Impact on surgery	May reduce unnecessary biopsies and support margin delineation	Reduction in number of MMS stages; potential margin reduction	No direct role	Full control over margins
Clinical role	Diagnosis + margin mapping (high-resolution superficial imaging)	Diagnosis + margin mapping	Screening/triage	Definitive diagnosis
Artefacts	Limited penetration-related artefacts; reduced visibility of deeper structures	False-positive margin extension due to imaging artefacts; shadowing and signal attenuation in hyperkeratotic lesions	Optical artefacts related to surface reflection; limited visualisation in hyperkeratotic lesions	Sectioning artefacts; tissue shrinkage; distortion during processing
Limitations	Limited depth (200–300 µm); difficulty assessing tumour margins in depth	Limited penetration depth (1.5–2 mm); high accuracy for tumour depth ≤ 1 mm; reduced accuracy and underestimation of invasion depth in lesions > 1 mm	No depth assessment; strong operator dependence	Time-consuming; requires tissue processing; not real-time

## Data Availability

Since this article is a systematic review, no new data were created during this study. All data used are taken from published articles. A complete list of databases providing access to the materials used is provided in the “Materials and Methods” section. A list of all sources used, as well as the cited materials, is provided in the “References” section.

## Supplementary Materials

The following supporting information can be downloaded at: https://www.mdpi.com/article/10.3390/cancers18101562/s1, File S1: Prisma 2020 Checklist [46].

## Abbreviations

The following abbreviations are used in this manuscript:

OCT	Optical coherence tomography
BCC	Basal cell carcinomas
SCC	Squamous cell carcinomas
KC	Keratinocyte carcinomas
NMSC	Non-melanoma skin cancer
CI	Confidence interval
AUC	Area under the curve
MMS	Mohs micrographic surgery
OR	Odds ratio
κ	Cohen’s kappa
RCM	Reflectance confocal microscopy
NOS	Newcastle–Ottawa scale
UV	Ultraviolet
LC-OCT	Line-field confocal optical coherence tomography
SD-OCT	Spectral-domain optical coherence tomography
PRISMA	Preferred reporting items for systematic reviews and meta-analyses
PROSPERO	International Prospective Register of Systematic Reviews
